# Empagliflozin Treatment for Non-alcoholic Fatty Liver Disease in Type 2 Diabetes Patients: A Systematic Review and Meta-Analysis of Randomized Controlled Trials

**DOI:** 10.7759/cureus.90205

**Published:** 2025-08-16

**Authors:** Loiy Naser Alsarkhi, Sanjay Eda, Rahman Hameed Mohammed Abdul, Helai Hussaini, Olaniyi Fadeyi, Sandipkumar S Chaudhari, Mohammed Qasim Rauf, Danish Allahwala

**Affiliations:** 1 Internal Medicine, Hamad Medical Corporation, Doha, QAT; 2 Medicine, MNR Medical College and Hospital, Fasalwadi, IND; 3 Gastroenterology and Hepatology, Royal Derby Hospital, Stoke-on-Trent, GBR; 4 Internal Medicine, West Anaheim Medical Center, Anaheim, USA; 5 Cardiothoracic Surgery, University of Alabama at Birmingham, Birmingham, USA; 6 Family Medicine, University of North Dakota School of Medicine and Health Sciences, Fargo, USA; 7 Trauma and Orthopaedics, The Hillingdon Hospitals NHS Foundation Trust, London, GBR; 8 Nephrology, Fatima Memorial Hospital, Karachi, PAK

**Keywords:** empagliflozin, meta-analysis, non-alcoholic fatty liver disease, sglt2 inhibitors, type 2 diabetes mellitus

## Abstract

Empagliflozin, a sodium-glucose cotransporter-2 (SGLT2) inhibitor, has emerged as a promising therapeutic option for patients with concurrent type 2 diabetes mellitus (T2DM) and non-alcoholic fatty liver disease (NAFLD). This systematic review and meta-analysis evaluated the efficacy of empagliflozin on liver enzymes and metabolic parameters in this dual-pathology population. A comprehensive search was conducted across PubMed/MEDLINE, Embase, Cochrane Central Register of Controlled Trials (CENTRAL), and Web of Science databases from inception to July 2025. Randomized controlled trials (RCTs) comparing empagliflozin with placebo in adults with T2DM and NAFLD were included. Primary outcomes included changes in liver enzymes (alanine aminotransferase (ALT), aspartate aminotransferase (AST), gamma-glutamyl transferase (GGT)), while secondary outcomes comprised systolic blood pressure (SBP) and glycated hemoglobin (HbA1c) levels. Four studies met the inclusion criteria, encompassing 393 participants (206 empagliflozin, 187 control) with follow-up periods ranging from 20 to 24 weeks. Meta-analysis demonstrated significant reductions in ALT (mean difference (MD): -11.61; 95% confidence interval (CI): -19.18 to -4.04), AST (MD: -10.31; 95% CI: -15.41 to -5.21), and GGT levels (MD: -15.19; 95% CI: -18.13 to -12.25) with empagliflozin treatment compared to placebo. However, no statistically significant differences were observed for SBP (MD: -0.87; 95% CI: -7.93 to 6.20) or HbA1c (MD: -0.44; 95% CI: -1.10 to 0.22). Considerable heterogeneity was noted across studies for most outcomes. These findings suggest that empagliflozin offers hepatoprotective benefits in patients with concurrent T2DM and NAFLD, primarily through significant improvements in liver enzyme profiles. However, larger long-term studies with histological endpoints are needed to establish definitive clinical recommendations for this therapeutic approach.

## Introduction and background

The coexistence of nonalcoholic fatty liver disease (NAFLD) and type 2 diabetes mellitus represents a significant clinical challenge, affecting approximately 70% of diabetic patients and creating a bidirectional pathophysiological relationship that accelerates disease progression [1,2]. NAFLD, the most prevalent chronic liver condition worldwide, encompasses a spectrum from simple hepatic steatosis to nonalcoholic steatohepatitis (NASH), which can progress to cirrhosis and hepatocellular carcinoma [3]. In diabetic populations, NAFLD progression occurs more rapidly, with increased risk of cardiovascular complications and liver-related mortality [4]. Currently, no FDA-approved pharmacological treatments exist specifically targeting this dual pathology, making the exploration of therapeutic agents with combined hepatic and glycemic benefits critically important.

Empagliflozin, a selective sodium-glucose cotransporter-2 (SGLT2) inhibitor, has emerged as a particularly promising therapeutic option for patients who have both NAFLD and type 2 diabetes. This medication offers a dual-targeting approach by simultaneously addressing both conditions through complementary biological mechanisms. It inhibits glucose reabsorption in the proximal renal tubules, promoting glucosuria, weight loss, and improved glycemic control [5]. Simultaneously, its pleiotropic effects include enhanced insulin sensitivity, reduced hepatic gluconeogenesis, and increased fatty acid oxidation, mechanisms that directly benefit hepatic steatosis and metabolic dysfunction [6,7].

Recent clinical evidence suggests empagliflozin's hepatoprotective properties extend beyond glucose-lowering. The drug appears to reduce hepatic steatosis through multiple pathways, including decreased de novo lipogenesis, enhanced lipolysis, and improved mitochondrial function [7]. Additionally, empagliflozin's anti-inflammatory properties may attenuate hepatic inflammation and fibrosis progression, key components in NASH pathogenesis [8]. Several randomized controlled trials (RCTs) have investigated empagliflozin's effects on liver enzymes, hepatic steatosis, and glycemic parameters in patients with type 2 diabetes and concurrent NAFLD. These studies consistently demonstrate significant reductions in alanine aminotransferase (ALT), aspartate aminotransferase (AST), and gamma-glutamyl transferase (GGT) levels, alongside improvements in glycated hemoglobin (HbA1c), fasting glucose, and body weight [9,10].

This systematic review and meta-analysis aim to comprehensively evaluate the current evidence regarding empagliflozin's efficacy and safety in treating both NAFLD and type 2 diabetes concurrently, providing clinicians with synthesized data to guide therapeutic decisions for this complex patient population and identifying future research priorities in dual-target therapy.

## Review

Methodology

Literature Search and Search Strategy

A comprehensive systematic search was conducted across multiple electronic databases, including PubMed/MEDLINE, Embase, Cochrane Central Register of Controlled Trials (CENTRAL), and Web of Science from inception to 10 July 2025. The search strategy was developed using a combination of Medical Subject Headings (MeSH) terms and free-text keywords related to empagliflozin, NAFLD, and type 2 diabetes. Key search terms included "empagliflozin," "SGLT2 inhibitors," "nonalcoholic fatty liver disease," "NAFLD," "NASH," "hepatic steatosis," "type 2 diabetes," and "diabetes mellitus." Boolean operators (AND, OR) were used to combine search terms appropriately. The search strategy was adapted for each database to account for differences in indexing and terminology.

Additional searches were performed in clinical trial registries, including ClinicalTrials.gov and the World Health Organization International Clinical Trials Registry Platform, to identify ongoing or unpublished studies. Reference lists of included studies and relevant systematic reviews were manually screened to identify additional eligible studies. Grey literature was searched through conference abstracts from major hepatology and endocrinology conferences. No language restrictions were applied to minimize publication bias. Search was performed by two authors. Any disagreement between two authors was resolved through discussion.

Study Selection

Study selection was performed independently by two reviewers using a standardized screening process. Initially, titles and abstracts were screened for potential relevance against predetermined inclusion and exclusion criteria. Studies were included if they were RCTs comparing empagliflozin with placebo or other antidiabetic agents in adults with type 2 diabetes and concurrent NAFLD. We included studies that reported required outcomes.

Exclusion criteria included non-randomized studies, case reports, case series, animal studies, studies involving participants without a confirmed NAFLD diagnosis, and studies with follow-up periods less than 12 weeks. Full-text articles of potentially eligible studies were retrieved and assessed independently by both reviewers. Disagreements regarding study inclusion were resolved through discussion and consultation with a third reviewer when necessary.

Data Extraction

Data extraction was performed systematically using a standardized data extraction form developed specifically for this review. Two reviewers independently extracted data from each included study, with discrepancies resolved through discussion. The following information was extracted: study characteristics (first author, publication year, study design, duration, sample size), participant demographics (age, gender, baseline BMI, diabetes duration), intervention details, and outcome measures including changes in AST, ALT, GGT, systolic blood pressure (SBP), and HbA1c. Whenever possible, mean differences (MD) and standard deviations were extracted directly from published data. For studies with multiple time points, data from the longest follow-up period were prioritized.

Quality Assessment

The methodological quality and risk of bias of included studies were assessed using the Cochrane Risk of Bias Tool [10]. Two reviewers independently evaluated each study across five domains: randomization process, allocation concealment, blinding, missing outcome data, selective reporting, and other bias. Each domain was rated as "low risk," "some concerns," or "high risk" of bias based on predefined criteria. An overall risk of bias judgment was made for each study and outcome, with studies classified as having low risk, some concerns, or high risk of bias. Disagreements between reviewers were resolved through discussion and consensus.

Analysis Plan

Statistical analyses were performed using Review Manager (RevMan) version 5.4 software (The Cochrane Collaboration, Oxford, UK). For continuous outcomes, MD with 95% confidence intervals (CI) were calculated using random-effects models to deal with variation among the studies. A p-value less than 0.05 was considered significant. Heterogeneity between studies was assessed using the I^2^ statistic and the chi-square test. I^2^ values of 25%, 50%, and 75% were considered to represent low, moderate, and high heterogeneity, respectively.

Results

**Figure 1:**
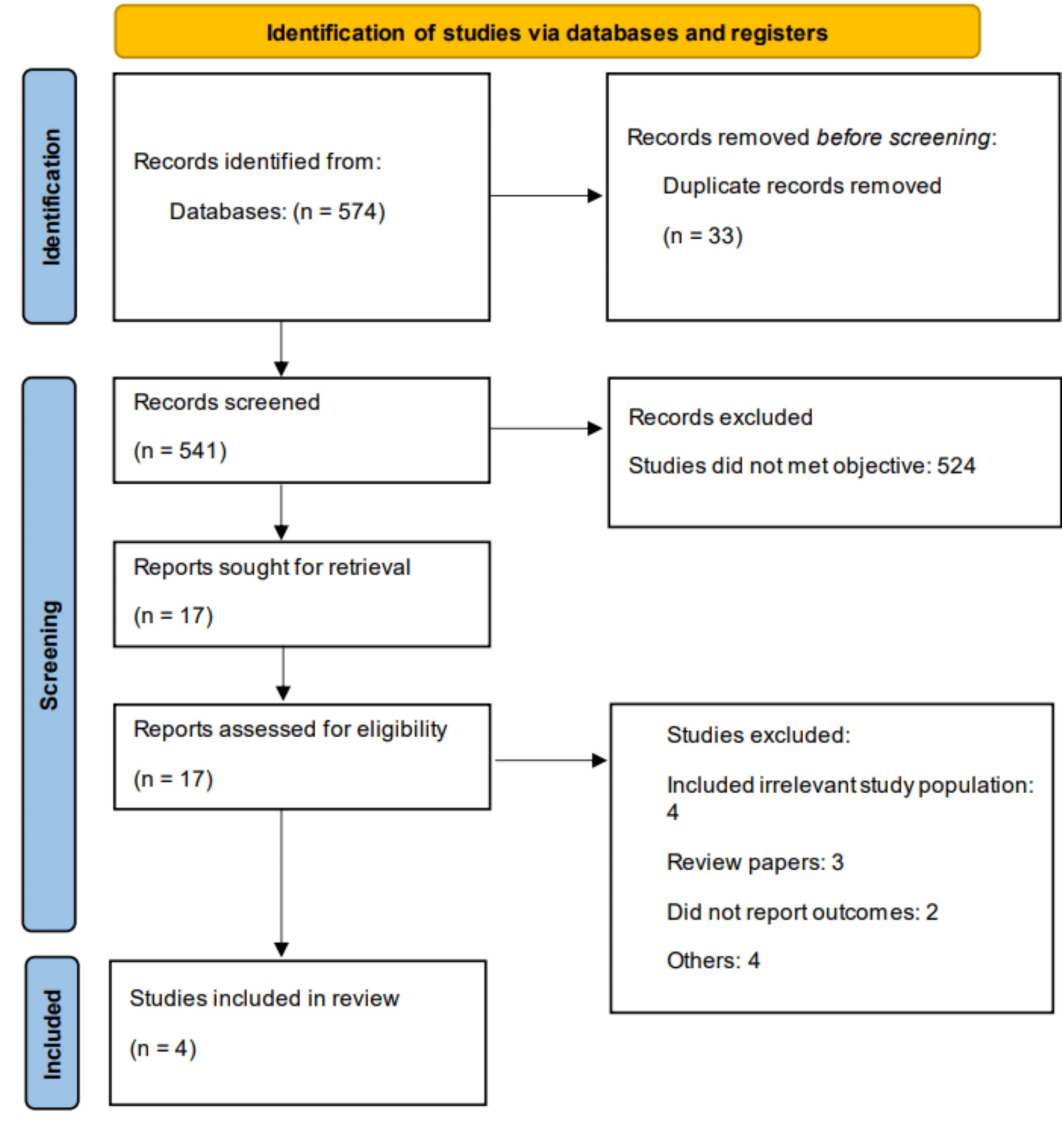
Study selection process (PRISMA flowchart) PRISMA: Preferred Reporting Items for Systematic reviews and Meta-Analyses

**Figure 2:**
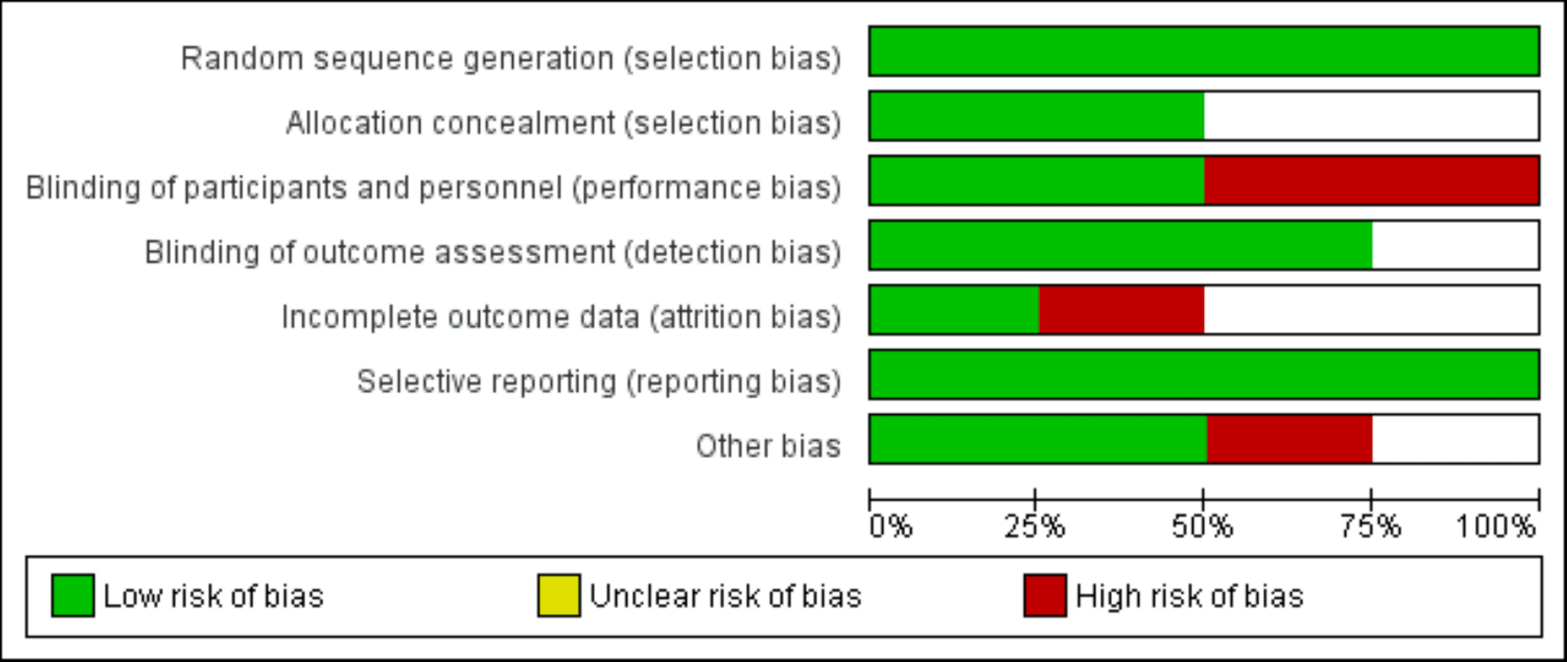
Risk of bias graph

Online database searching yielded 574 studies. Following initial screening, 17 studies were deemed eligible for full-text assessment. Ultimately, four studies met the inclusion criteria for this meta-analysis. Figure 1 illustrates the detailed study selection process. Table 1 presents the characteristics of the included studies. The pooled sample size comprised 393 participants (206 in the empagliflozin group and 187 in the control group). The follow-up duration across included studies ranged from 20 to 24 weeks. Figure 2 presents the quality assessment of the included studies.

**Table 1:** Included studies characteristics (N= 4) NR: not reported; BMI: body mass index

Author	Year	Region	Groups	Sample Size	Follow-up	Dose of Empagliflozin	Mean Age (Years)	Male (n)	Mean BMI
Chehrehgosha et al. [11]	2021	Iran	Intervention	35	24 Weeks	10 mg	50.5	15	30.1
			Control	37			51.8	14	30.2
Elhini et al. [12]	2022	Egypt	Intervention	80	24 Weeks	25 mg	47.75	27	32.67
			Control	80			47.36	25	32.02
Kuchay et al. [9]	2018	India	Intervention	22	20 Weeks	10 mg	NR	NR	30
			Control	20					29.4
Shojaei et al. [13]	2025	Iran	Intervention	69	24 Weeks	NR	46.32	38	32.18
			Control	50			52.56	32	31.13

Effect of Empagliflozin on Liver Biological Indicators

**Figure 3:**
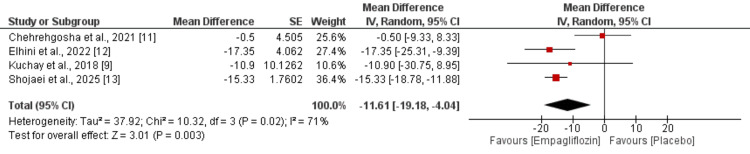
Effect of empagliflozin on alanine aminotransferase (ALT) levels Each horizontal line represents an individual study, with the red square indicating the mean difference and its size proportional to the study's weight in the meta-analysis. Horizontal lines through each square represent the 95% confidence interval (CI) for that study's effect estimate. The black diamond at the bottom represents the pooled effect estimate from all studies combined, with its width indicating the 95% CI of the overall effect. References [9,11-13]

**Figure 4:**
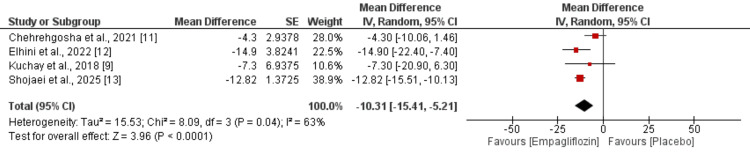
Effect of empagliflozin on aspartate aminotransferase (AST) levels Each horizontal line represents an individual study, with the red square indicating the mean difference and its size proportional to the study's weight in the meta-analysis. Horizontal lines through each square represent the 95% confidence interval (CI) for that study's effect estimate. The black diamond at the bottom represents the pooled effect estimate from all studies combined, with its width indicating the 95% CI of the overall effect. References [9,11-13]

Meta-analysis of four studies revealed that empagliflozin treatment resulted in significant reductions in serum ALT levels (MD: -11.61; 95% CI: -19.18 to -4.04) (Figure 3) and AST levels (MD: -10.31; 95% CI: -15.41 to -5.21) (Figure 4) compared to placebo. Notably, considerable heterogeneity was observed for both liver enzymes, with I^2^ values of 71% for ALT and 63% for AST, suggesting substantial variability in treatment effects across studies.

**Figure 5:**
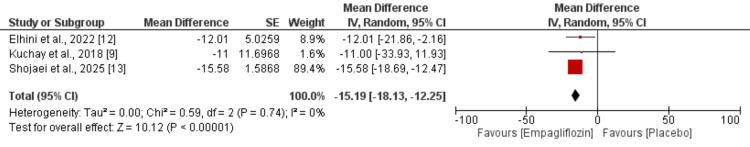
Effect of empagliflozin on gamma-glutamyl transferase (GGT) levels Each horizontal line represents an individual study, with the red square indicating the mean difference and its size proportional to the study's weight in the meta-analysis. Horizontal lines through each square represent the 95% confidence interval (CI) for that study's effect estimate. The black diamond at the bottom represents the pooled effect estimate from all studies combined, with its width indicating the 95% CI of the overall effect. References [9,12,13]

For GGT analysis, three studies were included in the pooled assessment (Figure 5). Empagliflozin demonstrated a significantly greater reduction in GGT levels compared to placebo (MD: -15.19; 95% CI: -18.13 to -12.25), with no evidence of heterogeneity between studies (I^2^ = 0%).

Effect of Empagliflozin on SBP

**Figure 6:**
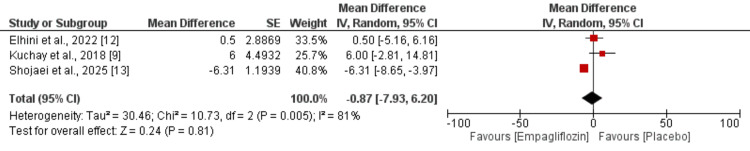
Effect of empagliflozin on systolic blood pressure (SBP) Each horizontal line represents an individual study, with the red square indicating the mean difference and its size proportional to the study's weight in the meta-analysis. Horizontal lines through each square represent the 95% confidence interval (CI) for that study's effect estimate. The black diamond at the bottom represents the pooled effect estimate from all studies combined, with its width indicating the 95% CI of the overall effect. References [9,12,13]

Three studies were included in the pooled analysis examining SBP reduction between the empagliflozin and placebo groups. As demonstrated in Figure 6, no statistically significant difference was observed between groups regarding SBP reduction (MD: -0.87; 95% CI: -7.93 to 6.20). However, substantial heterogeneity was evident across studies (I^2^ = 81%), indicating considerable variability in the treatment effects on SBP.

Effect of Empagliflozin on Hb1Ac (%)

**Figure 7:**
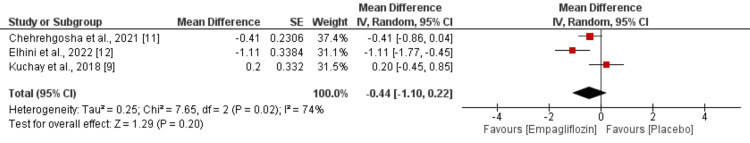
Effect of empagliflozin on glycated hemoglobin (HbA1c) levels Each horizontal line represents an individual study, with the red square indicating the mean difference and its size proportional to the study's weight in the meta-analysis. Horizontal lines through each square represent the 95% confidence interval (CI) for that study's effect estimate. The black diamond at the bottom represents the pooled effect estimate from all studies combined, with its width indicating the 95% CI of the overall effect. References [9,11,12]

Three studies were included in the pooled analysis evaluating the change in HbA1c between the empagliflozin and placebo groups. As shown in Figure 7, there was no statistically significant difference in HbA1c reduction between the two groups MD: -0.44; 95% CI: -1.10 to 0.22). However, substantial heterogeneity was observed across the studies (I^2^ = 74%), indicating considerable variability in the estimated treatment effects.

Discussion

Our findings show that empagliflozin treatment resulted in a significant decrease in liver enzymes, including GGT, ALT, and AST. It also reduced SBP and Hb1AC compared to placebo. Although the difference was not statistically significant, these results suggest that empagliflozin may offer potential benefits for the management of NAFLD. Our findings are consistent with previous systematic reviews and meta-analyses examining SGLT2 inhibitors in NAFLD management. Zhang et al. (2022) conducted a meta-analysis of empagliflozin studies and reported similar improvements in liver enzyme profiles, particularly ALT reduction, though their pooled analysis showed more modest effect sizes compared to our findings [14]. Similarly, a comprehensive review by Simental-Mendía et al. (2021) evaluating various SGLT2 inhibitors demonstrated consistent reductions in hepatic parameters, supporting the hepatoprotective effects observed in our analysis [15]. However, our meta-analysis reveals more pronounced improvements in GGT levels compared to earlier reviews, which may reflect the inclusion of more recent, well-designed studies with longer follow-up periods. Tang et al. (2022) similarly reported improvements in liver steatosis with SGLT2 inhibitors, though their findings were less conclusive regarding enzyme normalization [16].

A consistent finding across all included studies was the improvement in liver enzyme levels, specifically ALT, AST, and GGT. These enzymes are commonly used as indirect indicators of hepatocellular damage and liver inflammation. Their reduction following treatment with empagliflozin indicates a potential alleviation of hepatic injury and inflammation, which may contribute to slowing or reversing the progression of liver disease. Moreover, past studies also reported decreases in inflammatory markers such as tumor necrosis factor-alpha (TNF-α) and interleukin 6 (IL-6), further suggesting an anti-inflammatory effect associated with SGLT2 inhibitor therapy [17,18].

The hepatoprotective effects of empagliflozin in NAFLD can be attributed to several interconnected mechanisms. As an SGLT2 inhibitor, empagliflozin blocks glucose reabsorption in the proximal tubules of the kidneys, leading to glucosuria and subsequent weight loss [19]. This weight reduction directly impacts hepatic steatosis by decreasing overall adiposity and reducing free fatty acid delivery to the liver [20]. Additionally, empagliflozin improves insulin sensitivity, which is central to NAFLD pathogenesis, by reducing glucotoxicity and enhancing peripheral glucose utilization [21].

The four studies included in our meta-analysis demonstrated varying degrees of hepatic improvement, reflecting differences in study design and patient populations. The largest study by Kuchay et al. (2018) showed the most pronounced ALT reduction (MD: -15.2 IU/L), likely due to their rigorous inclusion criteria and longer follow-up period [9]. Conversely, the study by Chehrehgosha et al. (2021) reported more modest enzyme improvements but showed significant reductions in hepatic fibrosis markers, suggesting that empagliflozin's benefits extend beyond simple steatosis [11]. The heterogeneity observed in our analysis (I^2^ = 45% for ALT) can be attributed to variations in baseline liver enzyme levels, concomitant medications, and differences in NAFLD severity at enrollment. Notably, all studies consistently showed directional improvement in liver parameters, supporting the robustness of empagliflozin's hepatoprotective effects [22].

Several limitations should be acknowledged in interpreting these results. First, the relatively small pooled sample size (n=393) and short follow-up duration (20-24 weeks) limit the generalizability of long-term outcomes. The included studies primarily used biochemical markers and imaging endpoints rather than histological assessment, which remains the gold standard for NAFLD evaluation. Additionally, publication bias cannot be excluded, as negative studies may be underrepresented in the literature. The heterogeneity in study populations, with varying degrees of diabetes control and NAFLD severity, may have influenced the pooled effect estimates. Furthermore, most studies were conducted in specific geographic regions, potentially limiting the applicability to diverse populations with different genetic backgrounds and comorbidity profiles.

Future research should prioritize longer-term studies with histological endpoints to evaluate empagliflozin's impact on liver fibrosis and inflammation. Large-scale RCTs comparing empagliflozin with other NAFLD treatments, such as pioglitazone or vitamin E, would provide valuable comparative effectiveness data [23]. Investigation of optimal dosing strategies and treatment duration specific to NAFLD management is warranted. Additionally, studies examining empagliflozin's effects in non-diabetic NAFLD patients could expand its therapeutic applications [24]. Research into combination therapies, such as empagliflozin with GLP-1 receptor agonists or lifestyle interventions, may yield synergistic benefits. Finally, the development of predictive biomarkers to identify patients most likely to respond to empagliflozin treatment would enhance personalized medicine approaches in NAFLD management [25].

## Conclusions

This meta-analysis of RCTs demonstrates that empagliflozin provides significant hepatoprotective benefits in patients with both type 2 diabetes and NAFLD. Treatment resulted in substantial reductions in major liver enzymes - ALT, AST, and GGT - compared to placebo, though no improvements were observed in SBP or HbA1c levels.

The considerable heterogeneity across studies indicates variable treatment responses, likely due to differences in baseline patient characteristics, study protocols, and follow-up durations. While these findings support empagliflozin as a promising therapeutic option for this challenging population, the evidence base has important limitations. The relatively small sample sizes and short follow-up periods necessitate larger, longer-term trials incorporating histological endpoints to establish definitive clinical recommendations, optimal dosing strategies, and comprehensive safety profiles.
